# Supplementary material for: Two-year death prediction models among patients with Chagas Disease using machine learning-based methods
Source: PLoS Negl Trop Dis. 2022 Apr 14;16(4):e0010356. doi: 10.1371/journal.pntd.0010356 (PMC9041770; doi:10.1371/journal.pntd.0010356)
Supplement: S1 Table — (DOCX) [file pntd.0010356.s002.docx]

**S1 Table - Predictor variables initially considered in the analyzes and how they were worked**

| **Predictive Variables From the Interview** | **Question and Answer Options** |
| --- | --- |
| Age | Calculated in years from date of birth |
| Gender | Gender  Male  Female |
| Self-declared skin color | What is your color?  White (White)  Mixed/Black/Asian/Indigenous (Not White) |
| Literate | Can you read and write?  No  Yes |
| Marital status | What is your marital status?  Single/Widowed (with a stable union)  Married/Not married, but living with a partner (with a stable union) |
| Per capita income | Calculated by dividing family income (what is your family income?) by the number of people living in the household (how many people live in your house including you) |
| Smoker | Which of the following phrases best defines your habits regarding cigarette use?  I've never smoked/Have smoked, but don't smoke anymore (No)  I currently smoke (Yes) |
| Drinker | How many times, in the last 30 days, did you drink?  Didn't drink in the last 30 days/ Less than once a week/1 to 2 times a week (No)  3 to 5 times a week / Every day (Yes) |
| Physical activity | Do you practice physical activity?  No  Yes |
| High cholesterol | Did a doctor or health care professional say that you had high cholesterol?  No  Yes |
| Leishmaniasis | Has any doctor or health professional ever said that you had visceral or tegumentary leishmaniasis?  No  Yes |
| Diabetes | Do you have diabetes?  No  Yes |
| Coagulation related disease | Do you have a coagulation-related illness?  No  Yes |
| Kidney disease | Do you have kidney disease?  No  Yes |
| History of acute myocardial infarction | Have you ever had an acute myocardial infarction?  No  Yes |
| Arterial hypertension | Do you have arterial hypertension?  No  Yes |
| Thyroid related disease | Do you have thyroid-related disease?  No  Yes |
| Presence of comorbidity | Did a doctor or health professional say that you have or had any chronic illness?  No  Yes |
| Difficulty to swallow | Do you have or have you had difficulty in swallowing or a feeling of "food stuck" in the esophagus?  No  Yes |
| Esophageal enlargement | Has any doctor ever said that you have an enlarged esophagus on X-ray?  No  Yes |
| Usual to have 3 days without a bowel movement | Is it common for you to not have a bowel movement for 3 or more days?  No  Yes |
| Bowel enlargement | Has any doctor ever said that you have an enlarged bowel on X-ray?  No  Yes |
| Climbing stairs | Can you climb a flight of 8 steps or more without stopping?  No  Yes |
| Vertigo | Do you feel vertigo or dizzy?  No  Yes |
| Edema | Do you have edema or swelling in your feet in the morning?  No  Yes |
| Visible blood vessels in the neck | Have you ever noticed visible blood vessels in your neck in the mirror when sitting or standing? |
| Irregularity in the self-reported ECG | Has a doctor ever mentioned irregularities or problems on your ECG?  No  Yes |
| Permanent self-reported pacemaker | Do you have a permanent pacemaker?  No  Yes |
| Racing heart | Have you ever noticed your heart racing or beating differently?  No  Yes |
| Shortness of breath upon waking up | Have you ever woken up during the night with shortness of breath, unable to breathe?  No  Yes |
| Shortness of breath on exertion | Do you feel short of breath on exertion, for example: climbing stairs or climbing slopes?  No  Yes |
| Fainting | Have you ever had fainting spells or lost consciousness?  No  Yes |
| Self-perception of health | How would you rate your health today?  Good  Very good  Very bad  Average  Bad |
| Benznidazole | Have you ever taken a drug called ROCHAGAN or BENZNIDAZOLE?  No  Yes |

| **Predictor variables from Complementary Exams** | **Form worked in the ML model** |
| --- | --- |
| Quantitative PCR | Numerical |
| Heart rate | Numerical |
| Heart rate variability | Numerical |
| Corrected QT interval | Numerical |
| QRS complex duration | Numerical |
| Isolated Right Bundle Branch Block plus Left Anterior Fascicular Block | Categorical  Present/absent |
| Isolated Right Bundle Branch Block | Categorical  Present/absent |
| Complete left bundle branch block | Categorical  Negative/Positive |
| Atrial fibrillation | Categorical  Negative/Positive |
| Ventricular hypertrophy with ST-T segment abnormalities | Categorical  Negative/Positive |
| Pacemaker | Categorical  Present/absent |
| Pathological Q-waves | Categorical  Negative/Positive |
| Ventricular extrasystoles | Categorical  Negative/Positive |
| Low QRS voltage complex | Categorical  Negative/Positive |
| Categorized NT-proBNP  Different categorizations were tested, and the option with the highest predictive power was adopted (cut-off point of 300). | Categorical  <300/≥300 pg/dl |
