# Supplementary figures and images for: Two-year death prediction models among patients with Chagas Disease using machine learning-based methods
Source: PLoS Negl Trop Dis. 2022 Apr 14;16(4):e0010356. doi: 10.1371/journal.pntd.0010356 (PMC9041770; doi:10.1371/journal.pntd.0010356)

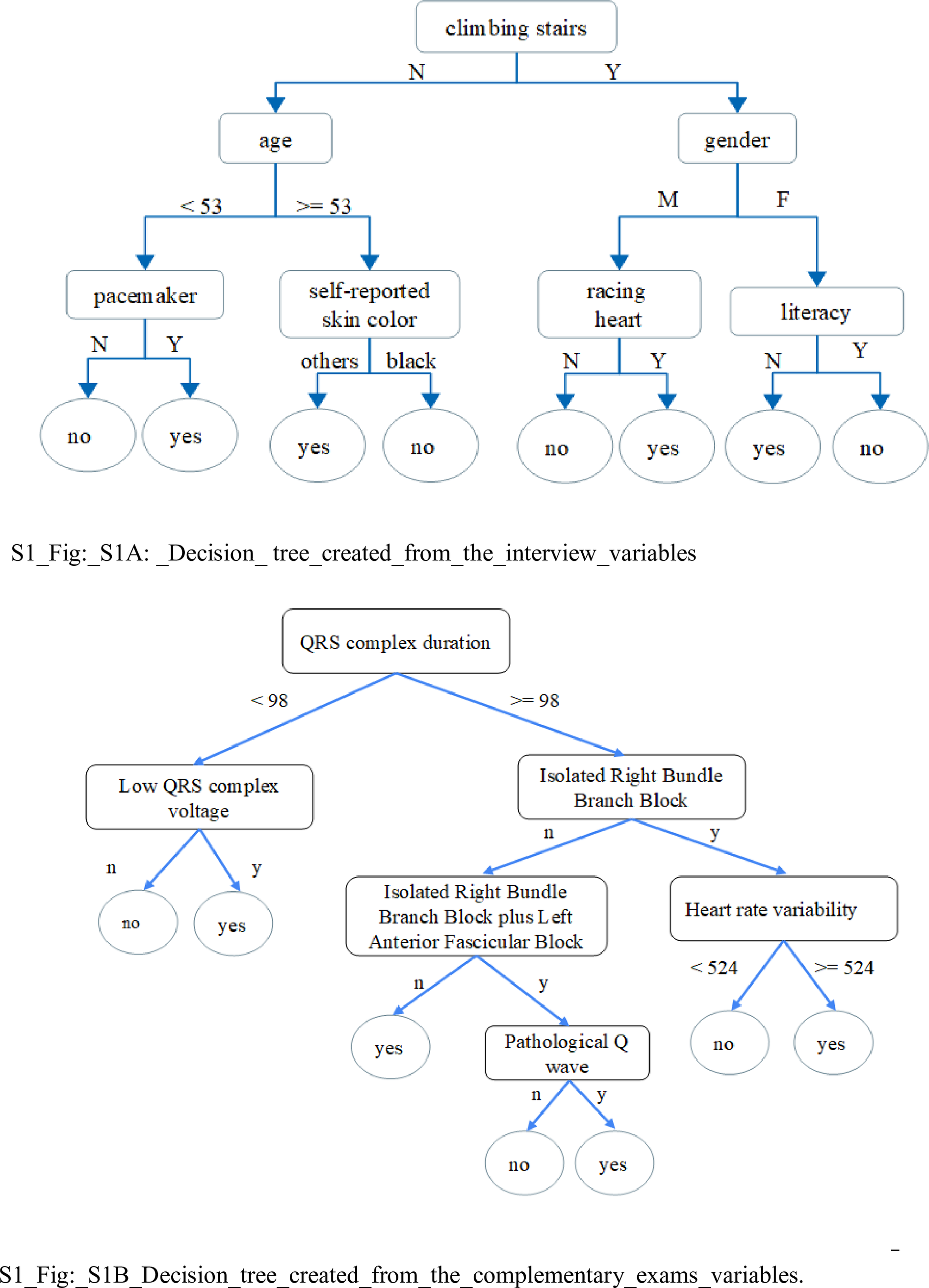

Supplement: S1 Fig — S1A: Decision tree created from the interview variables and S1B: Decision tree created from the variables of complementary exams. (TIF) [file pntd.0010356.s004.tif]
