# Supplementary material for: Two-year death prediction models among patients with Chagas Disease using machine learning-based methods
Source: PLoS Negl Trop Dis. 2022 Apr 14;16(4):e0010356. doi: 10.1371/journal.pntd.0010356 (PMC9041770; doi:10.1371/journal.pntd.0010356)
Supplement: Attachment — Submitted filename: PNTD-D-21-01555.pdf [file pntd.0010356.s005.pdf]

PLOS Neglected Tropical Diseases

Development of a two-year death prediction model among patients with Chagas Disease using methods based on machine learning

--Manuscript Draft--

**Manuscript Number:**	PNTD-D-21-01555
**Full Title:**	Development of a two-year death prediction model among patients with Chagas Disease using methods based on machine learning
**Short Title:**	Death prediction model among patients with Chagas Disease
**Article Type:**	Research Article
**Keywords:**	Chagas Disease, Forecasting, Machine Learning
**Abstract:**	Chagas disease (CD) is recognized by the World Health Organization as one of the thirteen most neglected tropical diseases in the world. More than 80 % of people affected by CD ~~in the world~~ will not have access to diagnosis and continued treatment, which supports the high morbidity and mortality rate. Machine Learning (ML) can identify patterns in data that can be used to increase our understanding of a specific problem or make predictions about the future. Thus, the aim of this study was to develop models and evaluate different ML techniques to predict death within two years, of patients with CD. Data from a cohort study with CD patients called SaMi-Trop and carried out in the state of Minas Gerais were analyzed. The predictor variables came from the baseline; and the outcome, which was death, came from the first follow-up. The outcome prediction was performed with three different configurations: only with interview variables, only with complementary exam variables, and finally, with interview variables and complementary exam variables. For each experiment we initially selected the 10 most important predictor variables. Five supervised ML techniques were used separately to predict the death of the cohort participants. All models were evaluated in terms of Sensitivity, Specificity and G-Mean. Among the 1,959 individuals with CD considered, 134 (6.8 %) died within two years of follow-up. Using the predictor variables from the interview, the different techniques achieved a maximum accuracy of 62 % in predicting death. Among the variables from complementary exams, there was a correct prediction of up to 78 %. In this model, the protagonism of NT-proBNP was evident, where it was possible to observe that the ML model using only this single variable reached 76 % accuracy. When excluding the NT-proBNP variable from the model only with complementary exams, the correct prediction achieved a maximum of 66 %. The model that mixed interview variables and complementary exams achieved a prediction accuracy of 76 %. Overall, the five ML settings adopted showed approximately similar values. The ML model with information coming only from the interview is useful in scenarios with little access to complementary exams, but the use of the complementary NT-proBNP exam in isolation can significantly improve the prediction.
**Additional Information:**	
**Question**	**Response**
**Financial Disclosure**<br><br>Enter a financial disclosure statement that describes the sources of funding for the work included in this submission. Review the submission guidelines for detailed requirements. View published research articles from *PLOS NTDs* for specific examples.<br><br>This statement is required for submission and **will appear in the published article** if	The funders had no role in study design, data collection and analysis, decision to publish, or preparation of the manuscript.

the submission is accepted. Please make sure it is accurate.<br><br>**Unfunded studies**<br><br>Enter: *The author(s) received no specific funding for this work.*<br><br>**Funded studies**<br><br>Enter a statement with the following details:<br>• Initials of the authors who received each award<br>• Grant numbers awarded to each author<br>• The full name of each funder<br>• URL of each funder website<br>• Did the sponsors or funders play any role in the study design, data collection and analysis, decision to publish, or preparation of the manuscript?<br>• **NO** - Include this sentence at the end of your statement: *The funders had no role in study design, data collection and analysis, decision to publish, or preparation of the manuscript.*<br>• **YES** - Specify the role(s) played.<br><br>* typeset	
**Competing Interests**<br><br>Use the instructions below to enter a competing interest statement for this submission. On behalf of all authors, disclose any competing interests that could be perceived to bias this work—acknowledging all financial support and any other relevant financial or non-financial competing interests.<br><br>This statement **will appear in the published article** if the submission is accepted. Please make sure it is accurate. View published research articles	The authors have declared that no competing interests exist.

Powered by Editorial Manager® and ProduXion Manager® from Aries Systems Corporation

from *PLOS NTDs* for specific examples.<br><br>**NO authors have competing interests**<br><br>Enter: *The authors have declared that no competing interests exist.*<br><br>**Authors with competing interests**<br><br>Enter competing interest details beginning with this statement:<br><br>*I have read the journal's policy and the authors of this manuscript have the following competing interests: [insert competing interests here]*<br><br>* typeset<br><br>This statement is required for submission and **will appear in the published article** if the submission is accepted. Please make sure it is accurate and that any funding sources listed in your Funding Information later in the submission form are also declared in your Financial Disclosure statement.	
**Data Availability**<br><br>Authors are required to make all data underlying the findings described fully available, without restriction, and from the time of publication. PLOS allows rare exceptions to address legal and ethical	Yes - all data are fully available without restriction

concerns. See the PLOS Data Policy and FAQ for detailed information.<br><br>A Data Availability Statement describing where the data can be found is required at submission. Your answers to this question constitute the Data Availability Statement and **will be published in the article**, if accepted.<br><br>**Important:** Stating 'data available on request from the author' is not sufficient. If your data are only available upon request, select 'No' for the first question and explain your exceptional situation in the text box.<br><br>Do the authors confirm that all data underlying the findings described in their manuscript are fully available without restriction?	
**Describe where the data may be found in full sentences. If you are copying our sample text, replace any instances of XXX with the appropriate details.**<br><br>• If the data are **held or will be held in a public repository**, include URLs, accession numbers or DOIs. If this information will only be available after acceptance, indicate this by ticking the box below. For example: *All XXX files are available from the XXX database (accession number(s) XXX, XXX.)*.<br>• If the data are all contained **within the manuscript and/or Supporting Information files**, enter the following: *All relevant data are within the manuscript and its Supporting Information files.*<br>• If neither of these applies but you are able to provide **details of access**	All relevant data are within the manuscript and its Supporting Information files

**elsewhere**, with or without limitations, please do so. For example:<br><br>*Data cannot be shared publicly because of [XXX]. Data are available from the XXX Institutional Data Access / Ethics Committee (contact via XXX) for researchers who meet the criteria for access to confidential data.*<br><br>*The data underlying the results presented in the study are available from (include the name of the third party and contact information or URL).*<br>• This text is appropriate if the data are owned by a third party and authors do not have permission to share the data.<br><br>* typeset	
Additional data availability information:	

Powered by Editorial Manager® and ProduXion Manager® from Aries Systems Corporation

Manuscript

Click here to access/download;Manuscript;Manuscript (Ingles).docx

Development of a two-year death prediction model among patients with Chagas Disease using methods based on machine learning

Short Title: Death prediction model among patients with Chagas Disease

Ariela Mota Ferreira[1], Laércio Ives Santos[2], Ester Cerdeira Sabino[3], Antonio Luiz Pinho Ribeiro[4], Léa de Oliveira Campos Silva[3], Renata Fiúza Damasceno[1], Marcos Flávio Silveira Vasconcelos D'Angelo[5], Maria do Carmo Pereira Nunes[4], Desirée Sant´Ana Haikal[1]

[1] Graduate Program in Health Sciences, State University of Montes Claros (Universidade Estadual de Montes Claros), Montes Claros, Minas Gerais, Brazil

[2] Instituto Federal do Norte de Minas Gerais, Montes Claros, Minas Gerais, Brazil

[3] Institute of Tropical medicine, University of São Paulo (Universidade de São Paulo), São Paulo, São Paulo, Brazil

[4] Department of Internal Medicine, Federal University of Minas Gerais (Universidade Federal de Minas Gerais), Belo Horizonte, Minas Gerais, Brazil

[5] Department of Computer Science, State University of Montes Claros (Universidade Estadual de Montes Claros), Montes Claros, Minas Gerais, Brazil

ABSTRACT

Chagas disease (CD) is recognized by the World Health Organization as one of the thirteen most neglected tropical diseases in the world. More than 80 % of people affected by CD ~~in the world~~ will not have access to diagnosis and continued treatment, which [illegible]pports the high morbidity and mortality rate. Machine Learning (ML) can identify patterns in data that can be used to increase our understanding of a specific problem or make predictions about the future. Thus, the aim of this study was to develop models and evaluate different ML techniques to predict death within two years, of patients with CD. Data from a cohort study with CD patients called SaMi-Trop and carried out in the state of Minas Gerais were analyzed. The predictor variables came from the baseline; and the outcome, which was death, came from the first follow-up. The outcome prediction was performed with three different configurations: only with interview variables, only with complementary exam variables, and finally, with interview variables and complementary exam variables. For each experiment we initially selected the 10 most important predictor variables. Five supervised ML techniques were used separately to predict the death of the cohort participants. All models were evaluated in terms of Sensitivity, Specificity and G-Mean. Among the 1,959 individuals with CD considered, 134 (6.8 %) died within two years of follow-up. Using the predictor variables from the interview, the different techniques achieved a maximum accuracy of 62 % in predicting death. Among the variables from complementary exams, there was a correct prediction of up to 78 %. In this model, the protagonism of NT-proBNP was evident, where it was possible to observe that the ML model using only this single variable reached 76 % accuracy. When excluding the NT-proBNP variable from the model only with complementary exams, the correct prediction achieved a maximum of 66 %. The model that mixed interview variables and complementary exams achieved a prediction accuracy of 76 %. Overall, the five ML settings adopted showed approximately similar values. The ML model

with information coming only from the interview is useful in scenarios with little access to complementary exams, but the use of the complementary NT-proBNP exam in isolation can significantly improve the prediction.
Keywords: Chagas Disease, Forecasting, Machine Learning

Author's Summary

Chagas disease (CD) is a public health problem despite the partial control of its transmission. Up to 30 % of infected people may have cardiac alterations, which are associated with a worse prognosis, with high mortality rates. One of the strategies that can be used to define interventions in order to reduce the impact of CD would be Machine Learning (ML). Thus, the aim of this study was to develop models and evaluate different ML techniques to predict death within two years, of patients with CD. We included 1,959 patients with CD, considering 21 municipalities in endemic regions in Brazil over a two-year period. Of these, 6.8 % died. Our study revealed that it is possible to develop ML models which allows the development of tools to predict death within two years, among patients with CD. The different techniques ranged from 62 % to 78 % prediction accuracy. Thus, we observed that ML can be used as a useful tool with the potential to contribute to the survival of patients with CD worldwide, by identifying patients with a higher probability of death.

INTRODUCTION

Chagas disease (CD) is recognized by the World Health Organization as one of the thirteen most neglected tropical diseases in the world, and is caused by the protozoan *Trypanosoma cruzi* (*T.cruzi*) and remains a public health problem despite the partial control of its transmission [1-3]. Most patients with CD remain in the “chronic indeterminate form”, defined as persistent asymptomatic infection without cardiac or gastrointestinal tract involvement [4]. However, up to 30 % of chronically infected people may have cardiac abnormalities, which is the most serious complication of CD [5]. This condition is associated with a worse prognosis, with higher mortality rates compared to other causes of heart failure [2,6].

CD was the leading cause of disability-adjusted life years (DALY) lost among all neglected tropical diseases [7]. Previous studies estimate that more than 80 % of people affected by CD in the world will not have access to diagnosis and continued treatment, which supports the high morbidity and mortality rate and the social cost of the disease [4, 8].

One of the strategies that can be used to define interventions in order to reduce the impact of CD would be Machine Learning (ML). ML is a field of Artificial Intelligence based on computational algorithms that allow computers to learn directly from data, without being explicitly programmed [9]. ML algorithms analyze large volumes of data represented by many characteristics (predictor variables) in reasonable time, and can handle complex relationships between data, which makes them as accurate or more accurate than human specialists in some situations [10]. Thus, ML can be defined as a set of tools and methods to identify patterns in data. These patterns can be used to increase our understanding of a specific problem or make predictions about the future. In these configurations, the goal is to find a model that better explains the dataset. Thus, we say that the algorithm learns from the data [11].

The aim of this study was to develop models and evaluate different ML techniques to predict death within two years, of patients with CD.

METHOD

Ethical approval

Ethical approval was granted by ethics committees (Research Ethics Committee of the Faculty of Medicine of the University of São Paulo - protocol number: 042/2012; Research Ethics Committee of the State University of Montes Claros - protocol number: 2,393,610; and the National Institutional Review Council (CONEP), number: 179,685/2012). All subjects agreed to participate in the study and signed an informed consent form before starting the collection.

Studied population

SaMi-Trop is a multicenter study funded by the National Institute of Health (NIH) (grant: P50AI098461-02 and U19AI098461-06) designed by scientists from four Brazilian public universities in the states of Minas Gerais and São Paulo, that established a cohort of carriers of CD, recruited in 21 municipalities that are endemic for CD in the state of Minas Gerais.

In this cohort, the baseline took place in 2013 and 2014 and included 2,161 participants. Subsequently the first follow-up was in the years 2015 to 2016 with the permanence of 1,709 participants. Details on the recruitment and methodologies of this cohort can be accessed in a previous study [12].

Data collection

Baseline and follow-up visits were carried out in the public health units of Primary Health Care, where participants were interviewed, had blood samples taken, and electrocardiogram (ECG) tests performed. The interview addressed sociodemographic issues, lifestyle habits, clinical history, CD treatment, physical activity, and quality of life. In the follow-up, among other questions, the reason for the patient's loss of follow-up was also addressed, where the alternatives were: death, giving up, and not being located.

The present investigation was conducted with data from the SaMi-Trop cohort, with the predictor variables coming from the baseline, and the outcome coming from the first follow-up.

Outcome

The outcome “Death” was adopted for this study (no *vs* yes), and was collected i.e., death being the reason of loss during follow-up.

Inclusion and configuration criteria for experiments

In this study, the prediction of the outcome “Death” was performed with three different configurations: only with interview variables, only with complementary exam variables, and finally, with interview variables and complementary exam variables. Of the 2,161 participants at baseline, 202 with negative or indeterminate serology for CD were excluded, thus all participants included in this study (n=1,959) had confirmed serology. In addition to these, in each configuration additional exclusions occurred due to loss of information (missing). Table 1 shows the number of lost participants, the total number of participants included, in addition to the percentage of death verified in each configuration.

Table 1: Number of participants excluded, included, and percentage of death verified in each type of configuration adopted (n=1,959).

Configuration type	Participants excluded (missing)	Participants included	Percentage of death (%)
Only with interview variables	54 (2.75 %)	1,905	6.88
Only with complementary exam variables	172 (8.77 %)	1,787	6.99
With interview and complementary exam variables	172 (8.77 %)	1,787	6.99

Data pre-processing

The predictor variables of interviews and complementary exams initially considered in the analyses, as well as the way in which they were collected and worked on, are presented in the supplementary material (Supplement 01). Among the predictor variables from the interview, 33 variables were considered and among the predictor variables from complementary exams, 15 variables were considered, including information from the ECG, and the test to assess heart failure (NT-proBNP) and CD (quantitative PCR).

To choose the NT-proBNP categorization, three measures were tested: numerical, categorized by age [13], and categorized with a cutoff point of $\geq$ 300 pg/dl, the latter being the format that had the best predictive power for the outcome.

S1 Table- Predictor variables initially considered in the analyzes and how they were worked

Selection of predictor variables for the model

For each experiment we initially selected the 10 most important predictor variables according to the Random Forests ranking, a strategy based on a previous study [14]. However, other simulations using predictor variables of specific interest were presented.

Machine Learning Approaches

Five supervised ML techniques were used separately to predict the death of the cohort participants. The techniques used were: Random Forests [15], Adaptive Boosting [16], Decision Tree [17], Support Vector Machine [18], and Artificial Neural Networks [19]. For training and evaluation

of these models, Matlab software version R2015b was used, with 5-fold cross-validation, of which three were used for training (training set), one to adjust the hyperparameters of each technique (validation set) and one to evaluate the performance of the models (test set) (Figure 1).

FIG. 1: Flowchart of the process of selecting predictor variables based on cross-validation analysis for predicting death among patients with CD within two years of follow-up.

Table 2 presents a brief description and the adjusted hyperparameters for each ML technique.

Table 2: Description of Machine Learning (ML) techniques adopted in the study.

ML Technique	Description	Adjusted hyperparameters
Random Forests	Uses an aggregate of decision trees from randomly selected instances and predictors. Each tree predicts a problem class and the model prediction is determined by majority vote.	Number of trees, number of sampled predictors, fraction of sampled data.
Adaptative Boosting	Uses an aggregate of decision trees from randomly selected instances and predictors in the first tree. From the second tree onwards, the selection of instances is made considering a probability proportional to the prediction error of the previous trees.	Number of trees, Learning Rate.
Decision tree	Recursively defined structure composed of decision nodes and leaf nodes. A decision node contains a test on some predictor and for each result of that test there is a link to a subtree. A leaf node corresponds to one of the problem classes.	Maximum number of subdivisions.
Support Vector Machine	Searches for a hyperplane that maximizes the distance between instances of two different classes. When the problem dealt with has only two predictors, this hyperplane is represented by a line, and with *n* predictors a hyperplane with *n* dimensions is needed to adapt to the data.	Kernel function.
Artificial Neural Networks	Simulates the way a human brain learns through artificial neurons. An artificial neuron takes input information from an external source and combines such inputs with non-linear operations producing a result based on the assimilated knowledge.	Number of neurons in the hidden layer, number of epochs and learning rate.

Class imbalance problem

In all the experiments performed in this study, there was an imbalance in the outcome classes. The number of participants with a positive outcome (death category) was approximately 7 %, while the number with a negative outcome (non-death category) was approximately 93 %. Thus, it was necessary to insert a balancing step for the training set. The Synthetic Minority Oversampling

Technique (SMOTE) method with k = 10 was used to balance the number of instances of the two classes. SMOTE performs a reduced sampling of the majority class and synthesizes new data points in the minority class [20].

Model performance evaluation

All models were evaluated in terms of Sensitivity, Specificity and G-Mean. In the context of this study, sensitivity measures the probability of the system predicting a death, given that the death actually occurred. Specificity measures the probability of the system predicting a non-death, given that the non-death actually occurred. The G-Mean equates sensitivity and specificity ($G - mean = \sqrt{(sensitivity * specificity)}$), measuring the balance between the classification performances in the majority and minority classes in problems with class imbalance.

RESULTS

Among the 1,959 individuals participating in this study, 134 (6.8 %) died within two years of follow-up. Table 3 presents categorical predictor variables selected among the 10 with the greatest predictive power in one or more configurations adopted in this study (final model), and their association with death. Quantitative PCR and heart rate variability (numerical variables) were also selected among the 10 variables with the greatest predictive power. The mean PCR in the “non-death” group was 553.45 parasites/ml and in the “death” group it was 645.38 parasites/ml (p=0.502). The mean heart rate variability between the “non-death” group was 310.24 ms and in the “death” group it was 475.97 ms (p=0.012).

Table 3 - Descriptive and bivariate analysis of categorical predictor variables selected among those with the greatest predictive power, and their association with death in patients with Chagas disease (CD). Minas Gerais, Brazil (n=1,959).

Interview variables	*Descriptive*	*Bivariate*		*P-value*$^{\pi}$
		**Death**		
		**No**	Yes	
	**n (%)**	**n (%)**	**n (%)**	
Sociodemographic				
Gender				
Male	644 (32.9%)	585 (90.8%)	59 (9.2%)	0.004
Female	1315 (67.1%)	1240 (94.3%)	75 (5.7%)	
Literate *				
No	860 (44.1%)	781 (90.8%)	79 (9.2%)	<0.001
Yes	1092 (55.9%)	1039 (95.1%)	53 (4.9%)	
Age				
Up to 60 years	1063 (54.3%)	1014 (95.4%)	49 (4.6%)	<0.001

Above 61 years	896 (45.7%)	811 (90.5%)	85 (9.5%)	
Self-declared color *				
White	426 (21.8%)	397 (93.2%)	29 (6.8%)	0.933
Non-white	1524 (78.2%)	1422 (93.3%)	102 (6.7%)	
Per capita income*				
Greater than R$ 356.33	774 (39.9%)	711 (91.9%)	63 (8.1%)	0.039
Less than R$ 356.32	1166 (60.1%)	1099 (94.3%)	67 (5.7%)	
**Signs and symptoms reported**				
Climb stairs *				
No	732 (37.6%)	653 (89.2%)	79 (10.8%)	<0.001
Yes	1214 (62.4%)	1159 (95.5%)	55 (4.5%)	
Self-reported ECG irregularity *				
No	744 (38.7%)	695 (93.4%)	49 (6.6%)	0.654
Yes	1180 (61.3%)	1096 (92.9%)	84 (7.1%)	
Racing heart *				
No	702 (36.5%)	649 (92.5%)	53 (7.5%)	0.404
Yes	1222 (63.5%)	1142 (93.5%)	80 (6.5%)	
**Comorbidities reported**				
Arterial hypertension				
No	706 (36.0%)	671 (95.0%)	35 (5.0%)	0.013
Yes	1253 (64.0%)	1154 (92.1%)	99 (7.9%)	
Permanent self-reported pacemaker *				
No	1804 (93.8%)	1691 (93.7%)	113 (6.3%)	<0.001
Yes	120 (6.2%)	100 (93.3%)	20 (16.7%)	
Variables from complementary exams				
Heart rate *				
Normal	1291 (67.6)	1203 (93.2%)	88 (6.8%)	0.136
Below normal (up to 59 bpm)	585 (30.6%)	550 (94%)	35 (6%)	
Above normal (above 101 bpm)	34 (1.8%)	29 (85.3%)	5 (14.7%)	
Corrected QT interval *				
Normal (up to 440 m/s)	947 (49.6%)	909 (96%)	38 (4%)	<0.001
Altered (above 441 m/s)	963 (50.4%)	873 (90.7%)	90 (9.3%)	
QRS complex duration *				
Normal (up to 120)	1117 (58.5%)	1069 (95.7%)	48 (4.3%)	<0.001
Altered (above 121)	793 (41.5%)	713 (89.9%)	80 (10.1%)	
Isolated right bundle branch block plus left anterior fascicular block *				
Negative	1692 (88.6%)	1584 (93.6%)	108 (6.4%)	0.121
Positive	218 (11.4%)	198 (90.8%)	20 (9.2%)	
Isolated right bundle branch block *				
Negative	1530 (80.1%)	1424 (93.1%)	106 (6.9%)	0.427
Positive	380 (19.9%)	358 (94.2%)	22 (5.8%)	
Pacemaker*Pacemaker				
Absent	1842 (96.4)	1732 (94%)	110 (6%)	<0.001
Present	68 (3.6)	50 (73.5%)	18 (26.5%)	
Pathological Q waves*				
Negative	1620 (84.8%)	1530 (94.4%)	90 (5.6%)	<0.001
Positive	290 (15.2%)	252 (86.9%)	38 (13.1%)	
Low QRS complex voltage *				
Negative	1799 (94.2%)	1687 (93.8%)	112 (6.2%)	
Positive	111 (5.8%)	95 (85.6%)	16 (14.4%)	
Categorized NT-proBNP *				
Normal (below 300 pg/dl)	1368 (70.2%)	1340 (98%)	28 (2%)	<0.001
Altered (above 301 pg/dl)	581 (29.8%)	475 (81.8%)	106 (18.2%)	

* Variation of the n = 1.959 because of missing information.

$^{\pi}$ Chi squared test

Prediction with interview variables

The 10 most important predictors from the interview (according to Random Forests ranking) were: age, literacy, per capita income, climbing stairs, self-reported ECG irregularities, self-reported skin color, self-reported permanent pacemaker, gender, arterial hypertension, and racing heart. The results of the 5 ML techniques are shown in Table 04. The best results in terms of sensitivity and G-Mean were from the Random Forests technique (the only technique that obtained both sensitivity and specificity above 0.60). In general, the different techniques revealed relatively similar and modest values, with a G-mean of approximately 60 % (0.60).

Table 04: Performance of models in predicting death for patients with CD, within two years, according to each machine learning technique adopted, considering the ten interview variables with the greatest predictive power.

	Specificity	Sensitivity	G-Mean
Random Forests	0.60	0.66	0.62
Adaptative Boosting	0.67	0.53	0.59
Decision Tree	0.64	0.58	0.60
Support Vector Machine	0.59	0.54	0.54
Artificial Neural Networks	0.68	0.52	0.59

Prediction with variables from complementary exams

The 10 most important predictor variables from complementary exams were: categorized NT-proBNP, QRS complex duration, heart rate variability, isolated right bundle branch block, quantitative PCR, corrected QT interval, low QRS complex voltage, heart rate, pathological Q Wave and isolated right bundle branch block plus left anterior fascicular block. Among them, it was observed that the predictive power of the NT-proBNP variable was greater (Figure 2).

Fig. 2: Importance of predictor variables of complementary exams for predicting death in patients with CD, within two years, according to Random Forests ranking.

The results of the 5 ML techniques considering the complementary exam variables are shown in Table 05. All techniques presented G-means above 73 %. Most models considering only

complementary exam variables presented predictive power superior to that achieved by models with the adoption of the ten interview variables (Table 5).

Table 05: Performance of models in predicting death for patients with CD, within two years, according to each machine learning technique adopted, considering the 10 variables of complementary exams with the greatest predictive power.

	Specificity	Sensitivity	G-Mean
Random Forests	0.76	0.76	0.76
Adaptative Boosting	0.76	0.79	0.78
Decision Tree	0.73	0.77	0.75
Support Vector Machine	0.73	0.79	0.76
Artificial Neural Networks	0.75	0.70	0.73

Due to the high predictive effect of the NT-ProBNP variable, two other models were tested: 1) excluding the categorized NT-proBNP variable (only the variables remaining: QRS complex duration, heart rate variability, isolated right bundle branch block, quantitative PCR, corrected QT interval, low QRS complex voltage, heart rate, pathological Q wave, isolated right bundle branch block plus left anterior fascicular block, and pacemaker); 2) Keeping this variable exclusively (Table 6 and 7, respectively).

Table 6: Performance of models in predicting death for patients with CD within two years, according to each machine learning technique adopted, considering the variables of complementary exams with greater predictive power, except NT-proBNP.

	Specificity	Sensitivity	G-Mean
Random Forests	0.65	0.67	0.66
Adaptative Boosting	0.67	0.66	0.66
Decision Tree	0.66	0.58	0.60
Support Vector Machine	0.62	0.72	0.66
Artificial Neural Networks	0.66	0.67	0.66

Table 7: Performance of models in predicting death for patients with CD within two years, according to each machine learning technique adopted, considering only the NT-proBNP variable

	Specificity	Sensitivity	G-Mean
Random Forests	0.74	0.79	0.76
Adaptative Boosting	0.74	0.79	0.76
Decision Tree	0.74	0.79	0.76
Support Vector Machine	0.74	0.79	0.76
Artificial Neural Networks	0.74	0.79	0.76

Prediction with interview and complementary exam variables

Using interview and complementary exam variables together, we tested whether there would be an improvement in the prediction of ML models. The results are shown in Table 08. Initially, the 20 most important predictor variables were included (10 from interviews and 10 from complementary exams) according to the Random Forests ranking. However, in the final model, 10 variables were kept: categorized NT-proBNP, QRS complex duration, heart rate variability, age, self-reported skin color, stair climbing, isolated right bundle branch block, quantitative PCR, corrected QT interval, and per capita income. In all techniques, no improvement in prediction was observed after using interview variables and complementary exams together. That is, models with only complementary exam variables (Table 04) had similar predictive power to models with complementary exam and interview variables (Table 08).

Table 08: Performance of models in predicting death for patients with CD within two years, according to each machine learning technique adopted, considering the ten interview variables and complementary exam variables with the greatest predictive power.

	Specificity	Sensitivity	G-Mean
Random Forests	0.79	0.68	0.73
Adaptative Boosting	0.73	0.79	0.76
Decision Tree	0.75	0.73	0.73
Support Vector Machine	0.71	0.78	0.74
Artificial Neural Networks	0.74	0.75	0.74

DISCUSSION

This study described how ML was able to predict death over a 2-year period in patients with CD, using different techniques and different configurations of predictive models. Using the 10 most important predictor variables from the interview, the different techniques revealed relatively modest values, with a maximum G-mean of 62 %. Using the 10 most important predictor variables from complementary exams, an increase in G-mean was observed, reaching up to 78 %. In this model, the role of NT-proBNP was evident (Figure 2), showing more than twice the importance observed for the other variables in the model. This finding was confirmed when observing the ML model using only NT-proBNP, where it was observed that this single variable reached a G-mean of 76 %. Other model configurations were also tested (with complementary exam variables excluding NT-proBNP and mixing variables from the interview and complementary exams). Even so, their predictive power was less than or equal to that verified by the model that considered NT-proBNP exclusively. In general, the five ML configurations adopted showed approximately similar values, and it was not possible to identify the superiority of one in relation to the others for all the adopted models, i.e., in each model, a different configuration reached a higher value. The prediction had good accuracy to recognize death with a good G-mean value (between 0.54 to 0.78), varying according to techniques and models.

In the prediction model with only variables from the interview, it was noticed that sociodemographic characteristics and self-reported signs/symptoms remained in the model. In this case, the high accuracy of self-reported questions for chronic conditions has already been verified [21]. In terms of clinical projection, adopting only ten interview variables during an anamnesis, individuals who would potentially benefit most from possible previous interventions would be identified. It would be possible to achieve 62 % accuracy in predicting death. Although the predictive power is considered relatively modest, it can still represent a great clinical impact. This intervention could be useful in practice, in places where there is restricted access to exams and specialized health services, with a "selective, focused and exclusive" offering [22]. The literature also reports that the groups that have greater health needs, such as those with CD, are precisely those that have greater difficulty in accessing and using health services [23]. It is a great challenge for the Brazilian public health service (SUS) to achieve equitable access, as each social segment has different demands produced by social processes of exclusion, not always perceived by the government [22]. Thus, the ML tool could contribute to this need for better management of health system users with CD, using simple and effective data, especially in remote regions.

In the prediction model only with variables from complementary exams, ECG, parasite load (quantitative PCR), and NT-proBNP biomarker variables remained. In this model, the prediction power achieved was greater than using only interview variables, demonstrating the importance of complementary tests in conducting the clinical management of CD. The ECG is the most important

complementary exam in the initial evaluation of patients with CD [4]. It is an inexpensive, standardized, and ubiquitous exam. Detection of *t.cruzi* parasites in blood by PCR has been used to assess the parasite burden and effectiveness of CD treatment, and previous studies have demonstrated the role of PCR in predicting CD progression [24, 25]. When verifying the importance of complementary exam variables to compose the model, the NT-proBNP biomarker was prominent. Thus, due to this importance, a model with only this variable was tested, and the predictive power of this model composed with this single variable was quite similar to the predictive power of the model with the 10 most important complementary exam variables (76 % *vs*. 78 %).

NT-proBNP levels are accurate discriminators of heart failure diagnosis, aid in patient risk stratification, and are powerful predictors of death. Their discriminatory ability to predict mortality (C = 0.69, 95 % CI: 0.66, 0.71) is similar to that of an ECG (C = 0.68, 95 % CI: 0.65, 0.71) [26]. Confirming this finding, NT-proBNP was included in a risk score to predict 2-year mortality in patients with CD, being the variable with the greatest power to predict death [27]. Furthermore, this measure is the factor most strongly associated with the occurrence of cardiovascular events in the population with CD [28].

Unfortunately, NT-proBNP is not readily available in most clinical settings involving CD. Its large-scale use in the Brazilian public health system as a routine test to aid in management is not envisioned at this time, although it is highly desired, because this exam, performed by a simple blood test, provides fewer echocardiograms (-58.2 %) and a reduction in the number of hospitalizations (-12.6 %). As it is also a strategy with lower final cost and better diagnostic accuracy, there would be no increase in the budget of the public health system for the diagnosis and treatment of patients with heart failure [29].

The model with complementary exam variables excluding NT-proBNP had 10 % lower predictive power (66 %). Despite this, given the restricted access to the NT-proBNP exam, this model (only with ECG and PCR data) can represent a useful tool.

In endemic areas, CD represents a major cause of death from cardiovascular disease [26]. A meta-analysis identified that CD is associated with high mortality, regardless of clinical condition, with a relative risk (RR) of 1.74 (95 % CI 1.49-2.03) and attributable risk of 42.5 % considering the exposed group [27]. The region where our study was conducted is characterized by having low demographic density, strong social inequality, large distances between municipalities, and extensive rural areas [30]. In addition to these contextual problems in the region, there is a lack of training, specific knowledge, and safety in the management of patients with CD among primary health care physicians [31, 32]. Therefore the use of ML tools can increase access to an adequate management of CD, considering that it has good results, and it is an automatic and inexpensive technique.

Among the strengths of this study, the longitudinal assessment of a large sample of patients with CD who live in endemic areas and in small municipalities, far from the large urban centers commonly portrayed in the studies, stands out. This makes it possible to extend the results to other similar locations, since the population with CD generally has a similar epidemiological profile [2]. But this study is not without limitations. First, it is important to highlight the discrete values for sensitivity and specificity of models based on interview variables. The second limitation is related to the way the models were evaluated in this study, using cross-validation to train and test the models. From a methodological point of view, cross-validation allows for a robust assessment. However, using two independent data sets (one for training and the other for testing the models) would better validate the generalizability of the models, making it possible to apply them in clinical practice. Implementation studies can be carried out to assess the potential impact of using these models to predict death in patients with CD.

CONCLUSION

It was possible to develop calibrated models, which allows the development of prediction tools. The ML model proved to be useful and with high power to predict death within two years among patients with CD. The different techniques ranged from 62 % to 78 % prediction accuracy. Models with information coming only from the interview have the advantage of being used in scenarios with little access to complementary exams, but the incorporation of variables from complementary exams has improved the predictive power. A model adopting only the NT-proBNP, in isolation, showed a prediction capacity similar to the best prediction models using interview variables and complementary exams. Thus, the ML technique confirmed the role of this biomarker in predicting death. ML can be used as a useful tool with the potential to contribute to the ~~survival~~ of patients with CD ~~worldwide~~, by identifying patients with the highest probability of death.

SUPPORTING INFORMATION LEGENDS

S1 Table: Predictor variables initially considered in the analyzes and how they were worked.
S2: Database

REFERENCES

1. Martins-Melo FR, Ramos Júnior AN, Alencar CH, Heukelbach J. Prevalence of Chagas disease in Brazil: a systematic review and meta-analysis. Acta Trop. 2014 Feb;130:167-74

2. Nunes MC, Dones W, Morillo CA, Encina JJ, Ribeiro AL. Chagas disease: an overview of clinical and epidemiological aspects. Journal of the American College of Cardiology. 2013;62(9):767-76. Epub 2013/06/19.

3. Ribeiro AL, Nunes MP, Teixeira MM, Rocha MO. Diagnosis and management of Chagas disease and cardiomyopathy. Nature reviews Cardiology. 2012;9(10):576-89. Epub 2012/08/01.

4. Nunes MCP, Beaton A, Acquatella H, Bern C, Bolger AF, Echeverria LE, et al. Chagas Cardiomyopathy: An Update of Current Clinical Knowledge and Management: A Scientific Statement From the American Heart Association. Circulation. 2018;138(12):e169-e209. Epub 2018/10/26.

5. Sabino EC, Ribeiro AL, Salemi VM, Di Lorenzo Oliveira C, Antunes AP, Menezes MM, et al. Ten-year incidence of Chagas cardiomyopathy among asymptomatic Trypanosoma cruzi-seropositive former blood donors. Circulation. 2013;127(10):1105-15. Epub 2013/02/09

6. Pereira Nunes M do C, Barbosa MM, Ribeiro AL, Amorim Fenelon LM, Rocha MO. Predictors of mortality in patients with dilated cardiomyopathy: relevance of chagas disease as an etiological factor. *Rev Esp Cardiol* 2010; 63(7): 788-97

7. Martins-Melo FR, Carneiro M, Ribeiro ALP, Bezerra JMT, Werneck GL. Burden of Chagas disease in Brazil, 1990-2016: findings from the Global Burden of Disease Study 2016. Int J Parasitol. 2019 Mar;49(3-4):301-310.

8. Borde Elis, Akerman Marco, Pellegrini Filho Alberto. Mapping of capacities for research on health and its social determinants in Brazil. Cad. Saúde Pública. 2014. Oct; 30 (10): 2081-2091

9. Minchole A, Rodriguez B. Artificial intelligence for the electrocardiogram. Nature medicine. 2019;25(1):22-3. Epub 2019/01/09.

10. Deo, Rahul C. Machine learning in medicine. Circulation, v. 132, n. 20, p. 1920-1930, 2015.

11. Wiens, Jenna; Shenoy, Erica S. Machine learning for healthcare: on the verge of a major shift in healthcare epidemiology. Clinical Infectious Diseases, v. 66, n. 1, p. 149-153, 2018.

12. Cardoso CS, Sabino EC, Oliveira CDL, Oliveira LC, Ferreira AM, Cunha-Neto E, et al. Longitudinal study of patients with chronic Chagas cardiomyopathy in Brazil (SaMi-Trop project): a cohort profile. BMJ Open. 2016; 6: e011181. doi: 10.1136/bmjopen-2016-011181 PMID: 27147390

13. Maisel A, Mueller C, Adams K Jr, Anker SD, Aspromonte N, Cleland JG, et al. State of the art: using natriuretic peptide levels in clinical practice. Eur J Heart Fail. 2008 Sep;10(9):824-39.

14. Genuer, Robin; POGGI, Jean-Michel; TULEAU-MALOT, Christine. Variable selection using random forests. Pattern recognition letters, v. 31, n. 14, p. 2225-2236, 2010.

15. Breiman, Leo. Random forests. Machine learning, v. 45, n. 1, p. 5-32, 2001.

16. Friedman, Jerome H. Greedy function approximation: a gradient boosting machine. Annals of statistics, p. 1189-1232, 2001

17. Breiman, Leo et al. Classification and regression trees. CRC press, 1984.

18. Cristianini, Nello et al. An introduction to support vector machines and other kernel-based learning methods. Cambridge university press, 2000.

19. Zurada, Jacek M. Introduction to artificial neural systems. St. Paul: West, 1992.

20. CHAWLA, Nitesh V. et al. SMOTE: synthetic minority over-sampling technique. Journal of artificial intelligence research, v. 16, p. 321-357, 2002.

21. Francisco Priscila Maria Stolses Bergamo, Azevedo Barros Marilisa Berti de, Segri Neuber José, Alves Maria Cecília Goi Porto, Cesar Chester Luiz Galvão, Malta Deborah Carvalho. Comparação de estimativas para o auto-relato de condições crônicas entre inquérito domiciliar e telefônico - Campinas (SP), Brasil. Rev. bras. epidemiol. [Internet]. 2011 Sep [cited 2018 Dec 04] ; 14(Suppl 1): 5-15.

22. Assis MMA, Villa TCS, Nascimento, MAA. Acesso aos serviços de saúde: uma possibilidade a ser construída na prática. Cien Saude Colet 2003;8(3):815-823.

23. Lima JC, Azoury EB, Bastos LHCV, Coutinho MM, Pereira NN, Ferreira SCC. Desigualdades no acesso e utilização de serviços de saúde no Brasil. Saúde Debate 2002;26(60):62-70

24. Basquiera A, Sembaj A, Aguerri A, Omelianiuk M, Guzmán S, Barral JM, Caeiro T, Madoery R, Salomone O. Risk progression to chronic chagas cardiomyopathy: Influence of male sex and of parasitaemia detected by polymerase chain reaction. Heart. 2003;89:1186-1190

25. Georg I, Hasslocher-Moreno AM, Xavier SS, Holanda MTd, Roma EH, Bonecini-Almeida MdG. Evolution of anti-trypanosoma cruzi antibody production in patients with chronic chagas disease: Correlation between antibody titers and development of cardiac disease severity. PLoS neglected tropical diseases. 2017;11:e0005796

26. Lima-Costa MF, Cesar CC, Peixoto SV, Ribeiro AL. Plasma B-type natriuretic peptide as a predictor of mortality in community-dwelling older adults with Chagas disease: 10-year follow-up of the Bambui Cohort Study of Aging. Am J Epidemiol. 2010 Jul 15;172(2):190-6.

27. Di Lorenzo Oliveira C, Nunes MCP, Colosimo EA, de Lima EM, Cardoso CS, Ferreira AM, de Oliveira LC, Moreira CHV, Bierrenbach AL, Haikal DSA, Peixoto SV, Lima-Costa MF, Sabino EC, Ribeiro ALP. Risk Score for Predicting 2-Year Mortality in Patients With Chagas Cardiomyopathy From Endemic Areas: SaMi-Trop Cohort Study. J Am Heart Assoc. 2020 Mar 17;9(6):e014176. doi: 10.1161/JAHA.119.014176. Epub 2020 Mar 11. Erratum in: J Am Heart Assoc. 2021 Feb 2;10(3):e014649. PMID: 32157953; PMCID: PMC7335521.

28. Ferreira AM, Sabino ÉC, Oliveira LCd, Oliveira CDL, Cardoso CS, et al. (2020) Impact of the social context on the prognosis of Chagas disease patients: Multilevel analysis of a Brazilian cohort. PLOS Neglected Tropical Diseases 14(6): e0008399

29. Araújo, DV; Souza CPR. Análise de Custo-Efetividade do Peptídeo Natriurético Tipo-B Comparado ao Julgamento Clínico em Pacientes com Dispnéia Aguda no Contexto do Sistema Único de Saúde. Rev SOCERJ. 2008;21(4):217-225 julho/agosto

30. Instituto Brasileiro de Geografia e Estatística (BR). Censo demográfico 2010. Rio de Janeiro; 2012.

31. Ferreira, AM; Sabino, EC; Moreira, HF; Cardoso, CS; Oliveira, CL; Ribeiro, ALP; Ramos, BC; Haikal, DS. Avaliação do conhecimento acerca do manejo clínico de portadores da doença de Chagas em região endêmica no Brasil. Rev. APS; 21(3): 345-354, 2018.

32. Damasceno RF, Sabino EC, Ferreira AM, Ribeiro ALP, Moreira HF, et al. (2020) Challenges in the care of patients with Chagas disease in the Brazilian public health system: A qualitative study with primary health care doctors. PLOS Neglected Tropical Diseases 14(11): e0008782.

Click here to access/download;Figure;FIG 1 correta (Ingles).docx

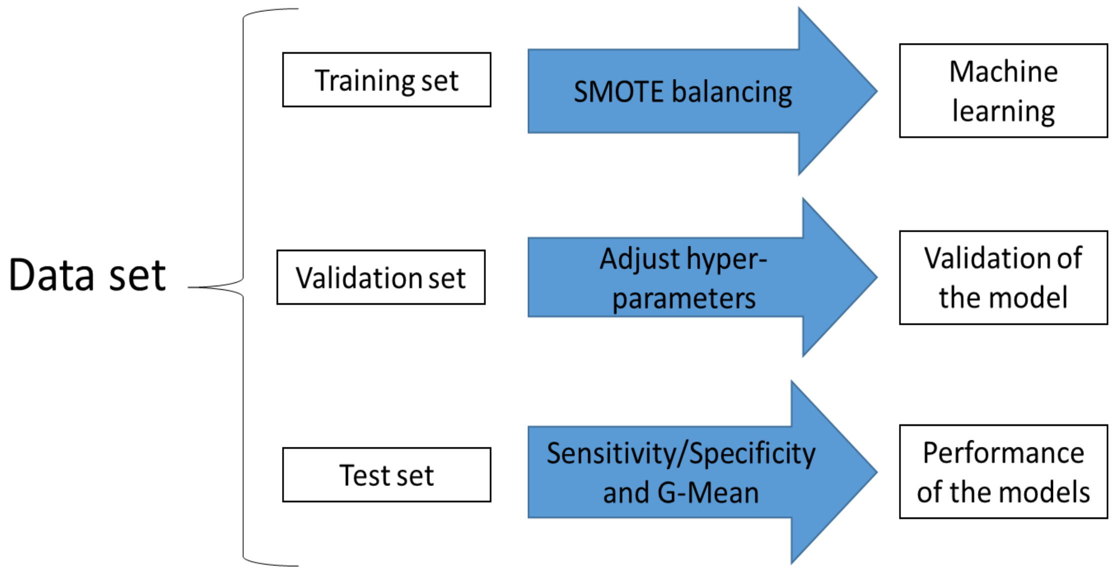

FIG. 1: Flowchart of the process of selecting predictor variables based on cross-validation analysis for predicting death among patients with CD in two years of follow-up.

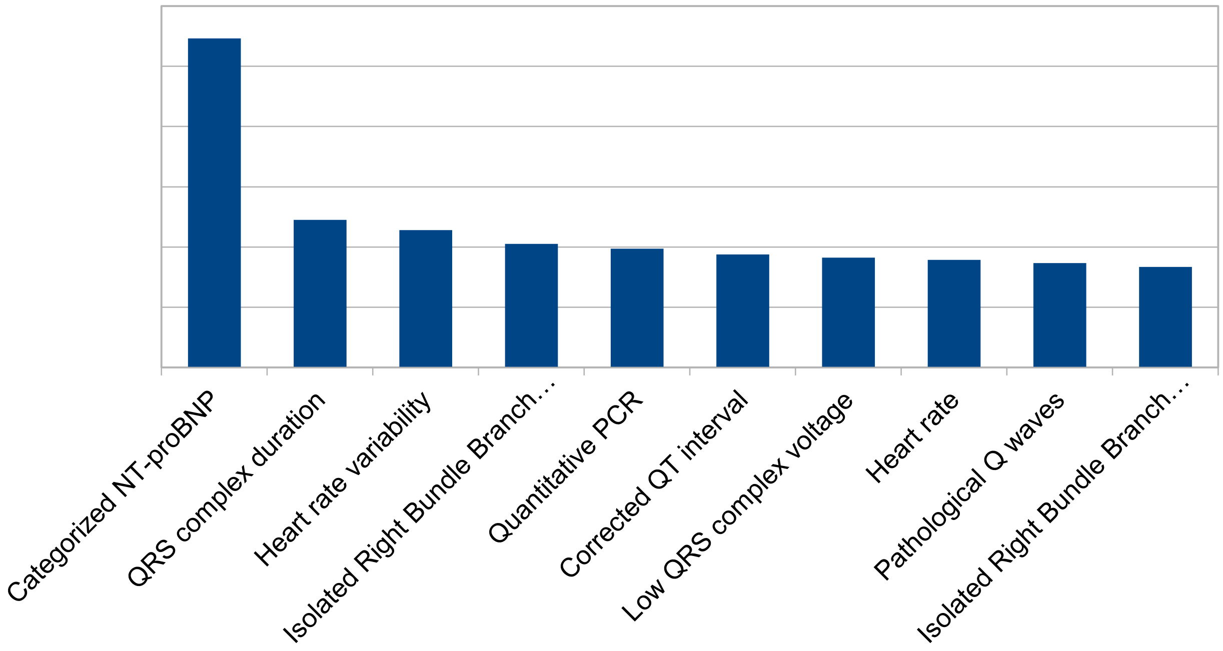

Fig. 2: Importance of predictor variables of complementary exams for predicting death in patients with CD within two years, according to Random Forests ranking.

S1: Table

Click here to access/download
Supporting Information
S1 Table.docx

S2: DAtabase

Click here to access/download
Supporting Information
banco_PLOS.xls
