# Supplementary material for: Two-year death prediction models among patients with Chagas Disease using machine learning-based methods
Source: PLoS Negl Trop Dis. 2022 Apr 14;16(4):e0010356. doi: 10.1371/journal.pntd.0010356 (PMC9041770; doi:10.1371/journal.pntd.0010356)
Supplement: Attachment — Submitted filename: carta_revisor.docx [file pntd.0010356.s006.docx]

**February, 1^th^, 2022.**

Dear Editor of Plos Neglected Disease,

Initially, on behalf of all the authors of this manuscript (PNTD-D-21-01555), I would like to thank both reviewers for all contributions you have made for our manuscript. We do believe that the criticisms and suggestions were fundamental for improvement of our study.

We have described the criticisms and suggestions reported by each reviewer followed by the author´s answers and comments about them. All changes that have been made in the manuscript by the authors have been highlighted in the text of the manuscript in order to facilitate their identification.

**Reviewer #1**

| The authors cite another paper initially describing the cohort from which the sample was driven. However, it forces the reader to go to this original paper to understand key points such as the inclusion and exclusion criteria and the manner in which these participants were recruited and included. I recommend the authors to clearly state, at least briefly, which were the inclusion and exclusion criteria, which diagnostic tests were performed in Chagas disease diagnosis work-up, and weather the participants were included sequentially in a period or randomly from the original cohort. | Thanks for the comment. The information was included in the “Studied population” section.  The period is described in lines 116-117 |
| --- | --- |
| Another issue in the method section is the very short follow-up period for Chagas disease patients. It is clear that this study reports from data of a partial follow-up not yet fulfilled, but I could not find any discussion regarding this short follow-up or expectations about the models’ behaviors with longer follow-up periods in the discussion section. Usually, Chagas Disease patients are followed for 10 or even more years for an outcome to be observed frequently, unless they are in a very advanced stage of (possible heart) disease. Therefore, it is also important to state in the inclusion and exclusion criteria if age or disease severity was considered for this purpose. | Thanks for the comment. The cohort was based on patients with cardiac alteration, this information was included in the manuscript (lines 115-116). |
| The outcome definition is extremely important for appropriate interpretation and applicability of the model. Therefore, I suggest the authors to clearly state if death (yes or no) is an overall CD death, overall heart CD death, any cause death, stroke related death, heart failure death, sudden death or any combination of these, or possible other mechanisms of death. These different mechanisms of death require different prevention and treatment approaches and may drastically change the applicability of the final model. | We acknowledge this limitation, but usually deaths associated with Chagas disease are due to cardiovascular causes, mainly sudden death or secondary to heart failure. In the present study, only 4 non-cardiovascular deaths occurred (one accidental death, two due to cancer and one non-specified death). However, as we were unable to assess the cause of death of each patient, all cause mortality was defined as the endpoint. This information has been added on line 132. |
| “In addition to these, in each configuration additional exclusions occurred due to loss of information (missing). Table 1 shows the number of lost participants, the total number of participants included, in addition to the percentage of death verified in each configuration.” seems result to me. I suggest moving this piece to results section. The method would be how to check missing data, how to decide on data imputation, and what methods are considered to impute data. Additionally, it is not clear what fraction of the excluded participants had the outcome. This could be easily cleared out rearranging table 1 or replacing it by an inclusion and exclusion diagram as recommend in TRIPOD. There is no comment on data imputation. Usually, missing data is not at random and analyzing complete cases only has potential to prediction bias, therefore complete case analysis is considered bad practice. Although there are several complex data imputation algorithms, such as predictive mean  matching and ML models, functions to make imputations are currently easy to use in several software, such as STATA and R-project. Of course, if missingness prevalence is very very low, imputation may seem pointless, however there is no way to know that (that Im aware of) until imputation is performed, and complete case data analysis is compared with imputed data analysis, but it does not seem to be the case. As seen in table one, two out of three scenarios have higher prevalence of participants with missing data then participants with the outcome. If missingness is not at random, i.e., it is strongly related to the outcome, then the outcome could be even doubled if missingness had never occurred in first place. How many of the participants with missing data have the outcome data missing? | Thanks for the the comment.  Table 1 received the outcome information (death) and was moved to the results section. Also, this data is in the flowchart as suggested.  Regarding missing data, in the literature there are reports of works that use two different approaches to deal with this problem: (1) exclude any record that has at least one variable with a missing value and (2) insert artificial values for missing values using some imputation strategy. The first strategy can lead to information loss and the second strategy can create unrealistic information in the dataset. However, a previous study advocates that a generally accepted guideline for managing missing data is that when they represent less than 10 % of the total information that must be present, strategy 1 could be used without a significant loss of statistical power in the data modeling results. As can be seen in Figure 2, in none of the configurations the percentage of missing data is greater than 10 %, thus, we understand that using the first strategy is a valid practice (CISMONDI et al., 2013).  Referências  CISMONDI, Federico et al. Missing data in medical databases: Impute, delete or classify?. Artificial intelligence in medicine, v. 58, n. 1, p. 63-72, 2013. |
| “To choose the NT-proBNP categorization, three measures were tested: numerical, categorized by age [13], and categorized with a cutoff point of ≥ 300 pg/dl, the latter being the format that had the best predictive power for the outcome.” Although there is reasoning for this procedure this statement is a bit awkward. There is plenty evidence pointing that categorizing continuous predictors is just loss of information and bad practice. By categorizing a continuous predictor in two classes, the authors are reducing the possibility of the machine learning techniques to identify complex data patterns which was mentioned as an advantage in the introduction section. In an extreme example, if BNP is an excellent predictor and may work alone replacing the whole model with a single decision threshold, why workout a machine learning algorithm in the first place? Additionally, similar comments can be worked towards all other predictors shown in table 3 such as PCR and income, and the outcome representation. It is not clear, but it seems that the machine learning techniques are representing the outcome as classes instead of probabilities as it would be expected in regression modeling. This modeling decision may also affect how the predictors may be combined and related to the outcome. Have authors tried regression ML instead of classes ML? Representing the outcome as classes turn the interpretation much easier but it hides the potential prediction error. If the predictions returns probabilities then discussions regarding calibration, decision thresholds and errors around this thresholds would be necessary | The focus of the work was more clinical, and in this case, the maintenance of the dichotomized form is supported in the literature [1, 2]. In addition, the variable was tested numerically and the model obtained was more fragile in terms of prediction power.  NT-proBNP was only identified as an excellent isolated predictor after training the ML algorithm. Initially, this predictive prominence of NT-proBNP was not known.  In the above example, where BNP is an excellent predictor and can work alone replacing the entire model, we thought it was important to train a machine learning algorithm because we also tested models with interview variables, and it was possible to reinforce the good predictive ability of NT-proBNP, we even provide a model without it.  We used the outcome variable in the form of classes instead of performing a regression, because this variable is naturally distributed in two classes in the data set used.   1. McCullough PA, Duc P, Omland T, et al. B-type natriuretic peptide   and renal function in the diagnosis of heart failure—an analysis from  breathing not properly (BNP) multinational study. Am J Kidney Dis March 2003;41(3):571–9.   2. Maisel A, Mueller C, Adams K Jr, Anker SD, Aspromonte N, Cleland JG, et al. State of the art: using natriuretic peptide levels in clinical practice. Eur J Heart Fail. 2008 Sep;10(9):824-39. |
| From the supplementary material it is clear that at least 45 predictors were available. Why did the authors chose to perform the models with ten predictors and not 8 or 6 or 12? What is the rationale involved in this decision? | We agree with the reviewer. In fact, there were a total of 45 predictor variables available (15 from complementary exam variables and 30 from interview variables). The models derived from complementary exam variables were evaluated with 5 and 10 variables, and the models with interview variables with 5, 10, and 15 variables. In all cases the configuration with 10 variables obtained the best results in terms of GMean and therefore was the chosen configuration. It is important to say that models using 10 variables obtained similar or superior results to models using all available variables in each configuration. |
| The following suggestion goes beyond the stated research aim. Instead of just comparing different machine learning techniques with binary outcomes the authors could try a comparison of the very same machine learning techniques with binary and survival outcomes. I believe there is a huge knowledge gap on this topic, it would be closer to the already available CD death prediction models in the literature and it would give a more elegant solution to the outcome missing data turning them into censored participants. Regarding the model’s performance measures I would suggest the authors to show the performances estimates from both test set and validation set. It is not clear how the subsamples for cross validation were defined. Where they splitted randomly? Or was there a geographic or calendar rationale? Additionally, a brief comment or discussion regarding the performance measures choices or alternatives, such as Youden index, accuracy, total error, positive likelihood ratio in predictive values would be nice. | There is a knowledge gap on the application of machine learning techniques to predict deaths from Chagas disease using non-parametric techniques with complementary interview and examination variables. We understand that this is one of the great contributions of this work.  The validation set is generally used to adjust the model's hyperparameters and its generalization capacity is measured in a third set, which in the case of this study, we call the test set. We believe that it is not necessary to show the performance estimates for this set, as this is not a common practice in this type of study and because we believe that this information will not add new knowledge to the discussion.  Cross-validation was performed in a stratified manner, that is, in each of the 5 subsets the prevalence of the outcome was preserved. This information was inserted into the text in the “Machine Learning Approaches” section.  Regarding performance measures, we emphasize that they are used, most of the time, in studies with the objective of validating diagnostic tests, which is not the case in our study. In addition, these measures can be calculated using sensitivity and specificity. |

**Reviewer #3**

| The authors present a manuscript of relevant interest. The subject falls within the scope of the journal. Overall, the paper is well written and contains valuable information. The bibliography is pertinent and current. However, the text still needs some improvement and minor repairs. Excerpts that deserve special attention in terms of writing were marked in yellow.Comments and additional suggestions have been placed in the margin of the text. | Thanks for the comment. The marked passages were reviewed and the suggestions accepted. |
| --- | --- |
| There are no data regarding the parasite load, a variable commented on and valued in the discussion. | Information on parasitic load can be found on lines 231-232. |
| Data for this variable are not in Table 8. | It was one of the variables that remained in the final model presented in table 8 |
| The importance of this variable (quantitative PCR) was not clearly presented and supported by the results and discussion. | Information on parasitic load can be found on lines 333-335 |
| Better substantiate the statement regarding PCR data | Information on parasitic load can be found on lines 333-335 |

**Results**

**Reviewer #1**

| What is the median follow-up time? What is the range of the follow up time? What is the fraction of participants loss to follow up or with interrupted follow-up? What is the rate of death expressed in events divided by person year (x 100 person-year)? Are participants living mainly in rural or urban areas? These are nice information to allow readers having an idea how the cohort behaves over time and compare with other Chagas disease cohorts. | Information was added in the “Studied population” section and in Figure 2. |
| --- | --- |
| Is it possible to show exclusion diagram as a result from the screening and recruiting effort? | Thanks for the comment. The suggestion was accepted. We have included Figure 2. |
| Regarding the subsamples for cross validation, it does not make sense estimating the outcome prevalence in all samples if they were splitted randomly, however as it is not clear how does samples were splitted the authors should consider to show the outcome prevalence from all samples. | Cross-validation was performed in a stratified manner, that is, in each of the 5 subsets the prevalence of the outcome was preserved. This information was inserted into the text in the “Machine Learning Approaches” section. |
| “In all techniques, no improvement in prediction was observed after using interview variables and complementary exams together. That is, models with only complementary exam variables (Table 04) had similar predictive power to models with complementary exam and interview variables (Table 08).”  What is the risk of death for a patient with altered and proBNP? How can a health care provider estimate a risk of death for his/her patient with any of these models? | The decision tree was built and inserted in the supplementary material 3.  The risk of death of a patient with altered BNP is cited in a previous study:  *Participants with a NT-proBNP levels > 300pg/ml had increased incidence of hard endpoint (hazard ratio (HR) (95% confidence interval (CI)): 3.62 (3.15–4.17) for all-cause mortality, and 6.38 (4.84–8.41) for cardiovascular mortality.*  Rudolf H, Mügge A, Trampisch HJ, Scharnagl H, März W, Kara K. NT-proBNP for risk prediction of cardiovascular events and all-cause mortality: The getABI-study. *Int J Cardiol Heart Vasc*. 2020;29:100553. Published 2020 Jun 5. doi:10.1016/j.ijcha.2020.100553 |

**Reviewer #2**

| The results are presented and discussed in accordance to the used methodology and some of its limitations have been highlighted. A number of qualitative conclusions are made, although little-to-none statistical evidence is provided to support them. Similarly, a number of unclear and subjective statements are made throughout the section regarding the statistical validity, robustness, and ability to generalise of the approach. From this perspective, a moderate use of definitive claims is recommended. The majority of the authors' claims are relevant, but only of qualitative nature and potentially questionable. | Thanks for the comment. We have revised and amended the entire text to comply with the suggestion.  In addition, we perform a statistical test that compares the results of the settings. We have inserted the subsection “Statistical analysis” at the end of the Methods section. |
| --- | --- |
| While it is true that it may be possible to use machine learning models in the clinical practice, it is mandatory that the relevance of the outcomes is properly scrutinised and their use in a real decision-making scenario conscientiously disciplined. I believe that strong statements regarding the validity of these methods and their results can only be made if a rigorous protocol is applied along the data collection and analysis pipeline. I am confident that the authors themselves would appreciate a more rigorous practice and degree of conscientiousness in the case they were the patients whose future chances of survival are predicted. | Thanks for the comment. We have revised and amended the entire text to comply with the suggestion.  It is not a robust enough model for real decision making in clinical practice.  More rigorous practice and a degree of conscientiousness in the case of patients whose future chances of survival are predicted is necessary.  There is an existence of a fundamental structure in the data and that is the strong point. |

**Reviewer #3**

| Some results are not clearly and completely presented. Some tables need minor repairs. | Thanks for the comment. The tables have been revised in order to make the information clearer. |
| --- | --- |

**Conclusions**

**Reviewer #1**

| “It was possible to develop calibrated models, which allows the development of prediction tools. The ML model proved to be useful…” There is no data supporting this conclusion. Neither in methods section nor in results section there is any mention regarding calibration statistics. Furthermore, it is known that machine learning techniques are usually bad calibrated models and there are several methods proposed to recalibrate the estimated probabilities from these techniques. However, it makes more sense to concern with calibration as a performance dimension with regression models, when probabilities are results of interest. Although the research has produced several models and one final model was considered, the authors did not present a prediction instrument from the final model, therefore stating that the models are useful is not supported by the results as it is. I can’t see how a reader or a health care provider can use any of such models to make predictions. I would expect a formula or a nomogram allowing users to make predictions. However, these are not applicable from machine learning techniques due to the “black box” phenomenon. A work around would be a web calculator. There are many many examples in the Internet of such tools, one I particularly find elegant is found in the following address: [https://breast.predict.nhs.uk/](https://breast.predict.nhs.uk/) and for chagas disease in the following address: [https://shiny.ini.fiocruz.br/pedrobrasil/](https://shiny.ini.fiocruz.br/pedrobrasil/) | Thanks for the comment. The entire manuscript was revised and unsupported terms for the conclusion were removed.  For a practical use of the study, there is still a need for the clinical implementation of this knowledge through the use of independent data for external validation. Therefore, a Decision Tree was developed to assist in future studies to validate the models. It is important to note that decision tree-based models are models that can be interpreted.  Supplementary material 3. |
| --- | --- |

**Reviewer #2**

| It is in my opinion difficult to make conclusive statements regarding the appropriateness of the study, as such statements should be based on the statistical properties of the sample and the complexity of the prediction model.  Because such information is only partially available or missing altogether, I can only assume that the models have been thoroughly optimized and that the class distribution was preserved in the creation of the 5 folds used for validating the hyperparameters. These are the minimal and essential requirements when dealing with statistical information: Their use is rigorously documented in the rich literature that modern public health has generated, it should not be neglected but rather integrated to support the use of machine learning techniques. | A statistical analysis of the data was performed comparing the results of the configurations.  This analysis is described in the subsection “statistical analysis” at the end of the methods section. The results are in the results section. |
| --- | --- |
| My opinion is that accuracies in the 70% range are not outstanding. However, and perhaps more importantly, they highlight the existence of a fundamental structure in the data which should be further discovered and explored. As it is a very uncommon in data analysis that a rigorous statistical treatment is developed. I advise the authors to consider a re-statement of the objectives of their work towards the usual practice to replace explicit statistical information with visual summaries of the data (typically, histograms and scatter plots), and support their presentation and the quality of nevertheless interesting investigations with highly valuable qualitative information. | Thanks for the comment. The entire manuscript has been revised to accommodate the suggestions. The results presentation format was changed to make the information more visual. |
| I encourage the authors to restructure their contribution with such summaries, as they would introduce the reader to the main features of the used data, rather than their questionable predictive power. | Thanks for the comment. The suggestion was accepted. |

**Reviewer #3**

| The discussion and conclusion related to the parasite load ( quantitative PCR) should be founded better. | Information on parasitic load can be found on lines 333-335. |
| --- | --- |
| The conclusion/discussion about the public health relevance should be reviewed ( see comment on line 353) | Thanks for the comment. The suggestion was accepted. |

**Editorial and Data Presentation Modifications?**

**Reviewer #3**

| The authors present a manuscript of relevant interest. The subject falls within the scope of the journal. Overall, the paper is well written and contains valuable information. The bibliography is pertinent and current. However, the text still needs some improvement and minor repairs. Excerpts that deserve special attention in terms of writing were marked in yellow. Comments and additional suggestions have been placed in the margin of the text. Minor remarks- Please see lines 22, 56, 68, 70, 197, 201, 212, 217, 234, 238, 248, 255, 256, 258, and 354. Major remarks- Please see comments on lines 252, 293-294, 321, and 353 | Thanks for the comment. We reviewed the entire manuscript and made the requested changes. |
| --- | --- |

**Summary and General Comments**

**Reviewer #1**

| It seems dad the authors where eager to develop prediction models with machine learning techniques. From my point of view, there could be at least two main approaches for this research. The first one would be to extensively test different ways to apply different machine learning techniques using Chagas disease as a case study scenario and discuss particular issues of the modeling process for Chagas disease data behavior with different models. Here, the model development and how to model would be of major interest. The second approach would be to really build a prediction instrument for different clinical scenarios of Chagas disease health care settings, in a way that health care providers could make predictions from these models. Here, the applicability of one or more models would be of major interest. Unfortunately, the authors fell in between those approaches, not fulfilling neither of those. To fulfill the first one, the suggestion would be to compare neural networks with binary outcome with neural networks with survival outcome, in a similar way to compare random forest with binary outcome with random forest with survival outcome etc. To fulfill the second approach the suggestion would be to construct a prediction instrument from the final model or from a couple of chosen models and make it available for users to make predictions. | In an attempt to fulfill the second approach, a decision tree was built (Supplementary Material 3). However, for a practical use of the study, there is still a need for the clinical implementation of this knowledge through the use of independent data for external validation. |
| --- | --- |
| It seems that no reporting guideline was followed as there are a few key point that I consider crucial for overall understanding that were poorly described. I would personally use the TRIPOD ([https://www.equator-network.org/reporting-guidelines/tripod-statement/](https://www.equator-network.org/reporting-guidelines/tripod-statement/)) as a reporting guideline, but there are others that could be as useful ([https://www.ncbi.nlm.nih.gov/pmc/articles/PMC7538196/](https://www.ncbi.nlm.nih.gov/pmc/articles/PMC7538196/)) | Thanks for the suggestion,we used the TRIPOD checklist. |
| Abstract: Chagas Heart Disease has three main mechanism of death which are: stroke, sudden death and heart failure. It is not clear which mechanism of death it is used as the outcome. If the overall mortality is the outcome, please state so. From the applicability point of view, it is important to know which mechanism of death are involved in the prediction as these mechanisms require different approaches regarding treatment and to courses of action in prevention. Additionally, overall mortality for chagas disease may have a substantial contribution from mortality not related to CD, as these patients live many years or even decades with this condition. Therefore, knowledge regarding this issue is important for appropriate interpretation and applicability of the final model. | We acknowledge this limitation, but usually deaths associated with Chagas disease are due to cardiovascular causes, mainly sudden death or secondary to heart failure. In the present study, only 4 non-cardiovascular deaths occurred (one accidental death, two due to cancer and one non-specified death). However, as we were unable to assess the cause of death of each patient, all cause mortality was defined as the endpoint. This information has been added on line 126. |
| The main objective of the research is to compare five different machine learning techniques, however in the abstract the authors do not state which techniques are being tested and compared. Please, state what are the models being compared. | The suggestion was accepted. |
| In abstract “Using the predictor variables from the 35 interview, the different techniques achieved a maximum accuracy of 62 % in predicting death…”. Is accuracy in this statement the same as overall correct classification or predictions? In the abstract, the authors stated that are three measures of interest to express the model’s performance, and accuracy is not one of them. Please define accuracy or state the model’s performance as one of the three measures of interest. | Thanks for the comment. We changed the suggested part. |
| Introduction: The authors state in the introduction that machine learning techniques learn from the data and can make predictions. Although I do agree with the authors, this also applies to regression models that are intended to prediction. The main characteristics of machine learning techniques that make it attractive over regression models are their automated procedures and the ability to capture complex patterns in the data in a way that implementing and updating the model in automated computerized systems make it much easier. However, this implementation does not seem to be the case, thus additional issues rise. Most if not all ML techniques suffer (more or less) from the “black box” phenomena. This means that if the data pilot inserts 30 predictors in a potential model, the algorithm shrinks the effect of the variables with no prediction contribution to zero without actually removing the predictor from the model. If the model is implemented in a way that the user needs to fill the 30 predictors values, it becomes less and less attractive to users as the number of predictors rises. Most of ML are just not made for removing the weak or near zero effect predictors, thus reducing the number of predictors is a workload balance between the data pilot effort and the user effort. This is briefly cited at the methods section “Selection of predictor variables for the model”. But the priorities of the authors are not clearly stated around this issue. From the performance point of view, there are plenty studies comparing performance of regressions and machine learning techniques with conflicting results. Additionally, recent systematic review points toward an evidence that there is no clear performance advantage off machine learning techniques over regression models when considering studies last susceptible to bias. Please take a look at the paper below  Christodoulou E, Ma J, Collins GS, Steyerberg EW, Verbakel JY, Van Calster B. A systematic review shows no performance benefit of machine learning over logistic regression for clinical prediction models. Journal of Clinical Epidemiology. junho de 2019;110:12–22. | Thanks for the comment. First, we improved the discussion around the benefits of variable selection in the “Selection of predictor variables for the model” section. As for the advantages that machine learning models have over regression models, it is important to say that models based on machine learning are able to find interactions between predictors without human intervention, deal better with problems that do not meet the linearity assumption, and can make consistent predictions even when there are a large number of predictors available. Furthermore, not all models used in this work are “black box” models. The decision tree shown in supplementary figure 3 is a model that can be interpreted relatively easily and quickly and even used to make predictions.    We can also highlight that, when working with regression models, some assumptions must be respected, such as the non-existence of high correlation between the predictor variables (in the case of logistic regression). However models derived from machine learning techniques are more flexible and do not require assumptions for their application. |
| The authors make no comment at all in the introduction about the usefulness or pitfalls of the models for death prediction for Chagas disease population already available in the literature. I suggest looking for models predicting death for Chagas disease, as I am aware of at least three of them, and comment about the potential improvement required in the field from this point on. | Thanks for the comment. Information was added in the introduction. |
| Methods: “Of the 2,161 participants at baseline, 123 202 with negative or indeterminate serology for CD were excluded, thus all participants included in 124 this study (n=1,959) had confirmed serology.” should be in the “study population” section. Often, it is confusing writing reports of studies that are actually sub studies mentioning a main study. Take special attention to mention key points applicable to substudy only or main cohort only. I suggest the authors clearly state which are the methods from the main cohort and the ones applied on this report. | Information on the cohort method was added to the manuscript. |
| Results: There is no scale value or axis label for the vertical axis at figure 2. Is it really supposed to be like this or there is something missing? | Thanks for the comment. We changed the figure to make it clearer. |
| Discussion: I believe there is two main results to be discussed from this research. the first one is the applicability of the chosen models which would be when and how these models could be used and alternatively when and how these models could not be used, or the limitations impairing their use. Additionally, how better or worse these models are when comparing to the existing models. This discussion is not present. The second main result is briefly commented in discussion. The authors stated that BNP alone is the strongest predictor. This is not exactly new as for long many researchers have shown that heart function expressed as ejection fraction is the is strongest predictor of death for this population. Additionally, it has a performance to identify death in two ears similar 2 many other combinations in the model and the authors cited a research where BNP was tested as prognostic marker showing similar performance to the observed in this research. So, what are the differences (advantages and disadvantages) of applying BNP as a single test, and applying BNP in the machine learning model? Of course, this discussion only makes sense if there is expectation to use BNP for new patients in the future. If this is not the case, it wouldn't make any sense to test BNP in a prediction model in first place. One point that should be in discussion section is what courses of actions are possible from these model results? What decisions health care providers can make or improve from the application of these models? Let's suppose a physician is making a prediction with one of these models and the model returns that this patient will die in the next two years. What could this physician do with this information? What are the future research to improve the results shown in this report and the field of Chagas disease mortality prevention?  Kind regards and may the force be with you. | Thanks for the comment. We have included a comparison with existing models in the literature in the introduction and discussion.  Regarding NT-proBNP, it was only identified as an excellent isolated predictor after training the ML algorithm. Initially, this predictive prominence of NT-proBNP was not known. Thus it can be part of the future external validation and support new studies. We thought it was important to train a machine learning algorithm because we also tested models with interview variables, and it was possible to reinforce the good predictive ability of NT-proBNP, even providing one without it.  For a practical use of the study, there is still a need for the clinical implementation of this knowledge through the use of independent data for external validation. Therefore, a Decision Tree was developed to assist in future studies to validate the models. It is important to note that decision tree-based models are models that can be interpreted.  Supplementary material 3 |
